# Cytoreductive Surgery (CRS) and Hyperthermic Intraperitoneal Chemotherapy (HIPEC): a Single-Center Experience in Austria

**DOI:** 10.1007/s11605-017-3661-1

**Published:** 2018-01-23

**Authors:** Kogler Pamela, Zitt Matthias, Kafka-Ritsch Reinhold, Punter Julia, Müssigang Peter, Perathoner Alexander, Öfner Dietmar

**Affiliations:** 1 Department of Visceral, Transplant and Thoracic Surgery, Medical University of Innsbruck, Anichstr. 35, 6020 Innsbruck, Austria; 2 Department of Surgery, Dornbirn Hospital, Dornbirn, Austria

**Keywords:** CRS, HIPEC, PSM, Morbidity, Mortality

## Abstract

**Background:**

Cytoreductive surgery (CRS) and hyperthermic intraperitoneal chemotherapy (HIPEC) can significantly influence overall and disease-free survival in selected patients suffering from peritoneal surface malignancies (PSM) of various tumor entities. Because of the extent of the therapeutic approach, the associated morbidity and mortality and the multidisciplinarity needed, implementation of a CRS + HIPEC program at an institution is often challenging.

**Methods:**

This single-center analysis included all patients (*n* = 60, 34 female, 26 male) with PSM from various tumor primaries [colorectal cancer (15/60; 25%), appendix neoplasia (21/60; 35%), and others (24/60; 40%)] treated with CRS + HIPEC at our institution between 2006 and 2014. Charts were reviewed for preoperative patient evaluation, procedure-specific and tumor-specific parameters, morbidity, mortality, tumor recurrence and patients’ overall (OS), and disease-free survival (DFS).

**Results:**

In 57 of the 60 patients included in the investigation (57/60; 95%), a radical resection (CC 0/1) was achieved. Median operating time was 559 min (253–900) with a median need of packed red blood cells of 1.1 (0–7) or fresh frozen plasma of 4.4 (0–20) concentrates. Twenty (33.3%) patients experienced 24 Dindo-Clavien grade III/IV complications (24/63; 38.1%). Postoperative 30- and 90-day mortality was 0% in our study population. Five-year OS was 43%, 5-year DFS 33%.

**Conclusions:**

Due to thorough preoperative patient evaluation, strict inclusion and exclusion criteria, and intense collaboration with other specialties, we were able to achieve an excellent 5-year OS of 43% with a CC score of 0/1 in 95% of our patient population. We were able to demonstrate the feasibility, efficacy, and safety of CRS + HIPEC in patients suffering from PSM at our institution.

## Introduction

Peritoneal surface malignancy (PSM) is defined as advanced or terminal stage of a tumor disease and depending on the tumor entity (e.g., gastric cancer) untreated PSM is associated with death within a brief period of time. Patients diagnosed with PSM in past decades were generally introduced to palliative systemic chemotherapy and/or palliative surgical procedures, especially when peritoneal carcinomatosis was diagnosed intraoperatively. Survival is poor at any rate: for example, median 15 months for PSM from colorectal primaries.^1^–^3^

Since the common assumption of PSM being a systemic metastatic disease changed to the concept of a localized tumor in the peritoneal cavity (now defined as a localized compartment), similar to the occurrence of liver-only metastasis, therapeutic strategies have changed dramatically over the last decade. Extensive research in this field evolved multimodal therapeutic approaches combining radical surgical procedures with perioperative and intraoperative chemotherapy with promising results.^4^–^8^ The liaison of complete cytoreductive surgery (CRS) and intraoperative hyperthermic intraperitoneal chemotherapy (HIPEC) became a feasible treatment option for several tumor entities with primary or secondary malignancies of the peritoneum (e.g. colorectal cancer, appendix neoplasms, pseudomyxoma peritonei, ovarian cancer). However, the CRS + HIPEC procedure is still mostly offered and realized at high-volume and academic centers, but not clinical routine everywhere (e.g., small volume hospitals): the reasons for this may be the high degree of technical effort, the lack of availability of this approach in most institutions, the small number of randomized controlled trials proving the benefit of this therapeutic regime and the extent of sophisticated multivisceral surgery with suspected higher mortality and morbidity rates.

In terms of pseudomyxoma peritonei (PMP) or peritoneal mesothelioma (PMT) CRS + HIPEC is the favored treatment regimen as this combined procedure can clearly prolong disease-free and overall survival. For PMP, median survival up to 196 months and a 10-year survival rate of 63% were achieved with CRS + HIPEC as compared to a 21% 10-year survival rate with CRS alone.^4^ For PMT, CRS + HIPEC can achieve 5-year survival rates of 47% with a median survival of 53 months^5^,^7^ in contrast to a median survival of 12 months with chemotherapy alone.^6^

In terms of PSM arising from a colorectal primary, CRS + HIPEC is close to finding its way into clinical routine as almost 30–40% of patients with colorectal primary develop peritoneal carcinomatosis without solid organ metastases.^8^–^11^ As mentioned above, median survival with chemotherapy and palliative surgery is poor, from seven to 24 months, even with the use of modern targeted therapies.^12^ CRS + HIPEC, by contrast, can increase median survival and achieve long-term survival in select patients. A multicenter registry study comparing CRS + HIPEC with sole systemic chemotherapy showed a median survival of 32 months and a 5-year survival of 27% after CRS + HIPEC in patients with PSM of colorectal origin.^13^ In patients with limited (low volume of carcinomatosis, defined as only two out of five abdominal regions affected) disease, this effect could even be amplified with a median survival of 63 months in the CRS + HIPEC group vs. 23.9 months in the modern systemic chemotherapy group.^12^

Important prognostic factors for the efficacy of CRS + HIPEC are the extent of the disease, measured by the peritoneal carcinomatosis index (PCI), and the completeness of cytoreduction.^14^

It is evident that CRS followed by HIPEC is a generally long and challenging procedure, often associated with multivisceral resections. Although these procedures can increase patient survival and improve outcome, morbidity and mortality can be high, particularly as CRS is combined with cytotoxic chemotherapy. Morbidity rates of 30–50% are published in the literature,^15^–^19^ with mortality rates of up to 12%.^20^ That said, standardized perioperative sequences with thorough patient selection, adequate infrastructure and a highly experienced surgical team are of essence to minimize morbidity and mortality and achieve best possible long-term outcomes.^15^,^19^,^21^

The goal of this study was to evaluate our single-center experience regarding feasibility, complexity and efficacy of CRS + HIPEC for PSM in terms of patient morbidity, mortality, and overall and tumor-specific survival. Additionally, we report here on our experience with the development and implementation of a PSM program at a university hospital in Austria.

## Patients and Methods

This retrospective analysis includes all patients surgically treated for PSM with CRS + HIPEC between June 2006 and December 2014, giving a total of 60 patients. Data were recruited from our computerized surgical documentation system, supplemented with data from our cancer follow-up program and the Cancer Registry of Tyrol. Collected information, such as patient characteristics, tumor diagnosis, histology, TNM staging and UICC classification, OP technique, HIPEC protocol, completeness of cytoreduction (CC score), morbidity and mortality, and recurrence and oncologic outcome, were entered in a database for statistical purposes (SPSS 24). This analysis was approved by the local institutional review board and was performed in accordance with the Helsinki Declaration of 1975, as revised in 1983.

### Preoperative Evaluation for CRS + HIPEC

All patients scheduled for CRS + HIPEC underwent accurate preoperative evaluation. Imaging techniques (e.g., CT, MRI, PET) were conducted to evaluate and select patients for CRS + HIPEC. The crucial inclusion criterion was histologically verified or explicit PSM at CT or MRI. Exclusion criteria were verified systemic metastatic spread (liver, lung) or spread to the pleural surface, age > 75 years or a World Health Organization performance status (WHO status) of ≥ 2. Furthermore, an imaging based PCI of > 20 in case of PSM of colorectal origin or > 10 in case of PSM from gastric cancer was defined as a contraindication for CRS + HIPEC and patients were introduced to other treatment modalities (e.g., palliative chemotherapy). Additional gynecologic or urologic examinations were performed preoperatively to further estimate the extent of the procedure (e.g., infiltration of the bladder, uterus, etc.). Furthermore, all patients planned for CRS + HIPEC were reviewed by our interdisciplinary tumor board.

### Surgical and HIPEC Procedure

After median laparotomy, the whole peritoneal cavity was explored in detail to evaluate the extent of tumor masses intraperitoneally. Liver metastases were ruled out by digital and, in the case of suspicious findings, intraoperative ultrasound examination. If complete tumor resection seemed possible, resection of the affected organs and peritoneum was performed. After complete (macroscopic) tumor resection, two inflow and two outflow cannulas were placed in the abdomen to ensure complete distribution of the chemotherapy solution. After placement of a temperature-tube at the base of the mesentery, the skin was provisionally closed with a running suture. In all patients, a “semi-closed” HIPEC technique was used. First, the abdominal cavity was rinsed with a heated solution (mulitBic®, ca. 5000 ml) until an “intraperitoneal” temperature of 42 °C was reached; second, the chemotherapeutic agent was added. A continuous inflow and outflow rate and a rotating patient position ensured equal distribution. After a defined time depending on the agent used (45–90 min), the chemotherapeutic solution was pumped out and the peritoneal cavity was again flushed with mulitBic®. The abdomen was opened again, cannulas were replaced and, after careful exploration, drains were put in place and the abdomen was closed with an absorbable running suture or interrupted non-absorbable sutures. HIPEC was performed with a modified heart-lung machine operated by a perfusionist.

The chemotherapeutic agent used for the HIPEC procedure was selected depending on the tumor entity and the performance status of the patient. We used oxaliplatin, mitomycin C, or cisplatin + doxorubicin according to the recommendations of the Austrian Society of Surgical Oncology (ASSO). Indications and dosages are presented in Table 2.

### Perioperative Systemic Therapy

Depending on the individual patient medical history, the diagnosed tumor entity and the occurrence of synchronous or metachronous PSM, the patient was given preoperative and/or postoperative systemic chemotherapy (CTX, e.g., gastric cancer, colon cancer) or radiochemotherapy (RCTX, e.g., anal cancer).

### Morbidity and Mortality

Perioperative complications were graded from 0–IV according to the Dindo-Clavien classification.^22^ Surgical site infections were classified according to the definition of the Center for Disease Control (CDC) as follows: SSI-1, superficial incisional infection; SSI-2, deep incisional infection; and SSI-3, organ/space infection. Mortality was defined as 30- and 90-day mortality after CRS + HIPEC.

### Statistical Analysis

Statistical analysis was performed with IBM SPSS 24 (Chicago, IL, USA). Statistical testing was based on appropriate techniques, depending on data distribution. Survival was analyzed with Kaplan-Meier procedures. Statistical significance was defined as *p* = 0.05.

## Results

### Patient and Tumor Characteristics

Of the 60 patients included in the study, 34 were female, 26 were male with a median age of 48 (range 13–74) years at PSM diagnosis. In total, 11 different tumor entities leading to PSM were included in the study (Table 1). Besides more common tumor entities like colorectal cancer (CRC) or appendix neoplasms, also rare entities like urothelial cancer or desmoplastic round cell tumors (DSRCT) were treated with CRS + HIPEC. PSM was synchronously diagnosed at the time of the primary tumor in 47 (47/60; 78.3%) patients, whereas 13 (13/60; 21.7%) patients developed PSM metachronously. With respect to the primary tumor, initial oncologic resection was performed at our surgical department in 42 (42/60; 70%) patients; 18 (18/60; 30%) patients were initially treated at an outside institution and transferred for CRS + HIPEC (Table 2).

**Table 1:** Tumor entities (%)

Colorectal cancer	15 (25)
Appendix neoplasia	21 (35)
Non-mucinous appendix-neoplasia	6
Mucinous appendix-neoplasia	14
LAMN	8
+ PMP	2
− PMP	6
MACA	
+ PMP	6
Neuroendocrine Appendix-Neoplasia	1
Gastric cancer	8 (13.3)
Ovarian cancer	4 (6.6)
Mesothelioma	3 (5)
DSRCT	3 (5)
Urothelial cancer	2 (3.3)
Pancreatic cancer	1 (1.7)
MANEC	1 (1.7)
Small bowel cancer	1 (1.7)
Anal cancer	1 (1.7)

*PMP* pseudomyxoma peritonei, *LAMN* low-grade appendiceal mucinous neoplasia, *MACA* mucinous adenocarcinoma, *DSRCTC* desmoplastic small round cell tumor, *MANEC* mixed adeno-neuroendocrine cancer

**Table 2:** Chemotherapeutic agent (dosage, time) + indication (*n*)

Cisplatin + doxorubicin (75 mg/m^2^ + 15 mg/m^2^ BSA; 60 min)	
GC	5
DSRCT	3
Ovarian Cancer	2
Mesothelioma	2
Urothelial Cancer	1
MANEC	1
Mitomycin C + cisplatin (30 mg/m^2^ + 50 mg/m^2^ BSA; 90 min)	
GC	3
CRC	1
Mitomycin C + doxorubicin (20 mg/m^2^ + 15 mg/m^2^ BSA; 60 min)	
Appendix carcinoma	1
Mitomycin C (30 mg/m^2^ BSA; 90 min)	
Anal Cancer	1
CRC	8
Small bowel cancer	1
Mesothelioma	1
Ovarian Cancer	2
Urothelial Cancer	1
Pancreatic Cancer	1
Epithelial appendix neoplasia	4
Mucinous appendix neoplasia	10
Neuroendocrine appendix neoplasia	1
Oxaliplatin (300 mg/m^2^ BSA; 45 min)	
CRC	6
Mucinous appendix neoplasia	4
Epithelial appendix neoplasia	1

*CRC* colorectal cancer, *PMP* pseudomyxoma peritonei, *LAMN* low-grade appendiceal mucinous neoplasia, *DSRCTC* desmoplastic small round cell tumor, *MANEC* mixed adeno-neuroendocrine cancer, *BSA* body surface area

### Perioperative Systemic Therapy

Seventeen out of the 60 patients (17/60; 28.3%) received preoperative systemic therapy, namely in 16 patients CTX [gastric cancer (*n* = 7), DSRCT (*n* = 3), non-mucinous appendix neoplasia (*n* = 3); CRC (*n* = 1), mucinous appendix neoplasia (*n* = 1), urothelial cancer (*n* = 1)] and in one patient RCTX (anal cancer). Twenty (20/60; 33.3%) patients were treated with solely postoperative adjuvant therapy [CRC (*n* = 8), mucinous appendix neoplasia (*n* = 4), non-mucinous appendix neoplasia (*n* = 3), MANEC (*n* = 1), neuroendocrine cancer of the appendix (*n* = 1), ovarian cancer (*n* = 2), and pancreatic cancer (*n* = 1)]. In five (5/60; 8.3%) patients, systemic therapy was given pre- and postoperatively [DSRCT (*n* = 2), urothelial cancer (*n* = 1), gastric cancer (*n* = 2)].

### Performance of CRS + HIPEC

In 57 of the 60 (57/60; 95%) study patients, a radical resection with complete resection of all macroscopically visible tumor nodules (CC 0/1) was achieved; in three (3/60; 5%) patients the extent of the PSM did not allow complete cytoreductive surgery (CC score > 1). To realize complete cytoreductive surgery, in 40 (40/60; 66.7%) patients a multivisceral resection (defined as resection of three or more different organs) had to be performed. In 54 patients (54/60; 90%), a bowel-resection was performed; in 9 patients (9/60; 15%), an ostomy (transient = 1, permanent = 8) had to be created.

In total, in over 8 years CRS + HIPEC procedures were performed by six consulting surgeons, two (median, range 1–4) of these surgeons were present at each operation including teaching operations. If an interdisciplinary operation was necessary (e.g. hysterectomy or partial resection of the urinary bladder), a gynecologist or urologist participated in the operation. Median operating time was 559 min (253–900) or 9.3 h (4.2–15). Intraoperative bleeding was generally rare with a mean packed red blood cell (PRBC) transfusion requirement of 1.1 (0–7) concentrates. Coagulation disorders called for fresh frozen plasma (FFP) transfusions in 32 (32/60; 53.3%) patients with a mean of 4.4 (0–20) concentrates. Mean hospital stay was 17.5 days (6–45); 30-day readmission rate due to postoperative complications was 5% (3/60).

### Morbidity and Mortality Following CRS + HIPEC

Out of the sixty patients, 35 (35/60; 58.3%) experienced 54 complications overall (Table 3). Itemized following the Dindo-Clavien classification, 20 (20/60; 33.3%) patients experienced 24 grade III/IV complications, mostly pleural effusions (7/60; 11.7%) or postoperative hemorrhage (6/60; 10%). Complications requiring surgical care emerged in 11 (11/60; 18.3%) patients due to intraabdominal abscess (SSI-3) (4/60; 6.7%) or postoperative hemorrhage (5/60; 8.3%). One patient developed an anastomotic leak (1/60; 1.7%) requiring surgical revision, one patient was again admitted to the operating room due to a ureter leak. Grade I complications were seen in 12 patients, grade II in three patients. Grade I complications were generally superficial wound infections (SSI-1) or postoperative prolonged ileus, grade II mostly neurogenic micturition malfunctions due to the extent of CRS. Itemized for duration of the CRS + HIPEC procedure, patients experiencing any type of complication had a longer median operating time of 617 min (253–900) compared to 545 min (315–800) in patients without complications.

**Table 3:** Morbidity of 63 procedures (CRS + HIPEC)

Morbidity	*n*	%
Surgical site infection (SSI)	15	23.8
SSI-1	10	15.9
SSI-3	5	7.9
Pleural effusion	7	11.1
Prolonged postoperative ileus	6	9.5
Postoperative intraabdominal hemorrhage	6	9.5
Line sepsis	3	4.8
Pneumonia/aspiration	3	4.8
Neurogenic micturition malfunction	2	3.2
Pancreatic fistula	2	3.2
Dermatologic reaction	2	3.2
Transitory kidney failure (need for dialysis)	1	1.6
Ureter leackage	1	1.6
Duodenal ulcus	1	1.6
Anastomotic leak	1	1.6
Transient multiorgan failure	1	1.6
Chylus fistula	1	1.6
Pneumothorax	1	1.6
Pulmonary embolism	1	1.6
Combined grade III/IV morbidity	24	38.1

Postoperative 30-day mortality was 0% in our study population. With respect to tumor-related cause of death, 90-day mortality was again 0%. One patient died within 90 days after CRS + HIPEC due to an iatrogenic perforation during upper gastrointestinal endoscopy for duodenal ulcer at an outside institution, giving an overall 90-day mortality of 1.7% (1/60).

### Oncologic Outcome

At the end of the investigational period of median 15.5 (range 0–110) months 31 (31/60; 51.7%) patients were considered tumor-free, five (5/60; 8.3%) patients were diagnosed with tumor progression and 23 (23/60; 38.3%) patients had died [CRC (*n* = 6), mesothelioma (*n* = 2), non-mucinous appendix neoplasia (*n* = 5), DSRCT (*n* = 2), GC (*n* = 5), ovarian cancer (*n* = 2), urothelial cancer (*n* = 1); one (1/60; 1,7%) patient was lost to follow-up.

Of the 31 patients considered tumor-free, 23 (23/31; 74.2%) patients had synchronous [CRC (*n* = 4), non-mucinous appendix neoplasia (*n* = 1), mucinous appendix neoplasia (*n* = 12), DSRCT (*n* = 1), gastric cancer (*n* = 2), mesothelioma (*n* = 1), ovarian cancer (*n* = 1), MANEC (*n* = 1)] and eight (8/31; 25.8%) patients metachronous [CRC (*n* = 2), non-mucinous appendix neoplasia (*n* = 2), anal cancer (*n* = 1), urothelial cancer (*n* = 1), small bowel cancer (*n* = 1), pancreatic cancer (*n* = 1)] PSM at the time of CRS + HIPEC. Of the five patients with recurrent disease, three (3/5; 60%) patients showed isolated distant recurrences (liver, lung, spleen), whereas two (2/5; 40%) patients developed a distant and peritoneal recurrent disease. Cause of death was tumor-related in 20 of the 23 (20/23; 87%) patients; one patient died because of a complication following upper gastrointestinal endoscopy (perforation), in two patients cause of death could not be determined. Interestingly, three patients with recurrent PSM [gastric cancer (*n* = 1), CRC (*n* = 1), mesothelioma (*n* = 1)] after CRS + HIPEC during the study period were again introduced to a surgical approach combined with HIPEC for curative intent. Of these, one patient was considered tumor-free at the end of the investigation period (mesothelioma), one patient was lost to follow-up (CRC), and one patient died (gastric cancer) due to tumor progression.

**Fig. 1:**
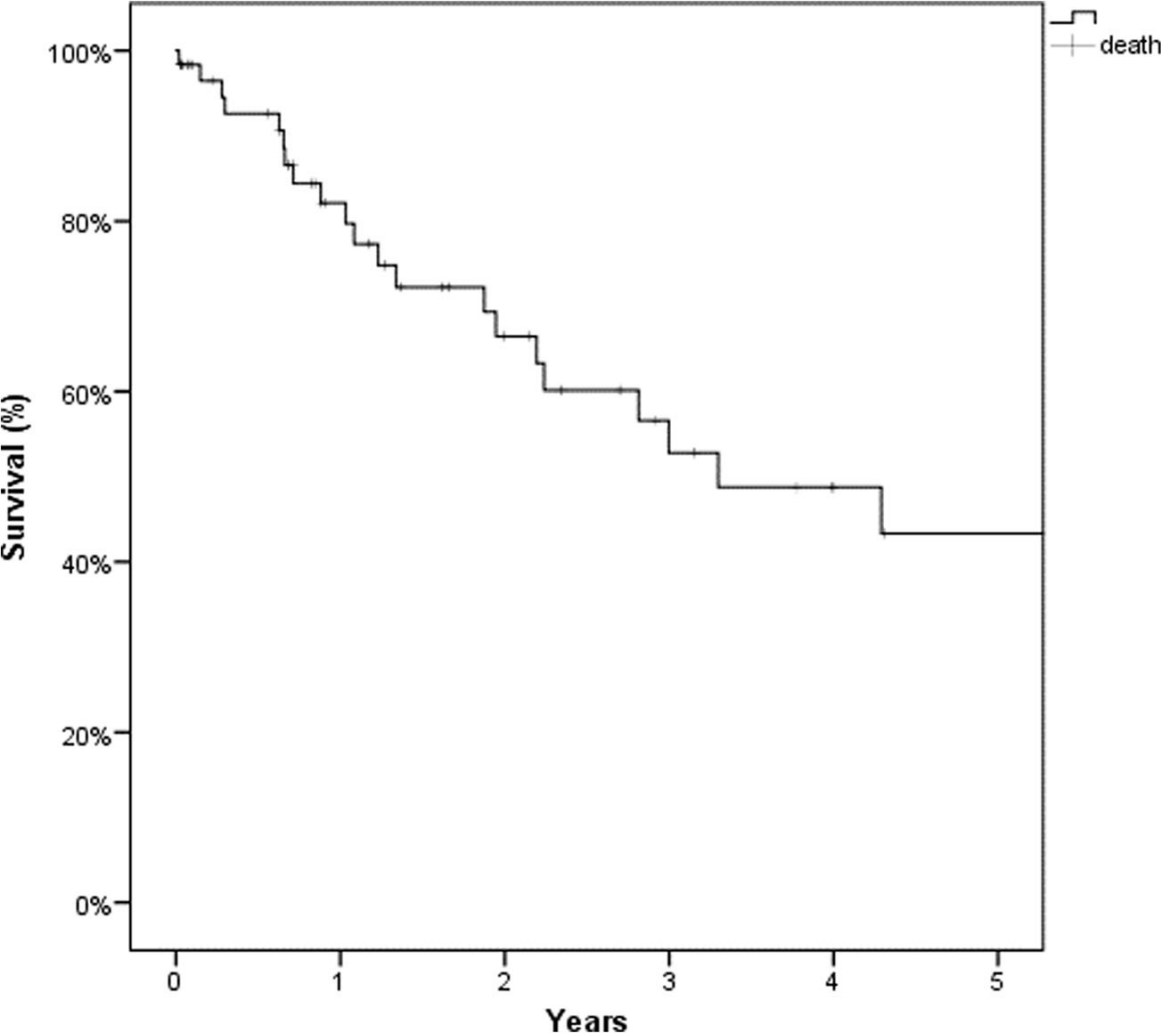
Overall survival (%)

**Fig. 2:**
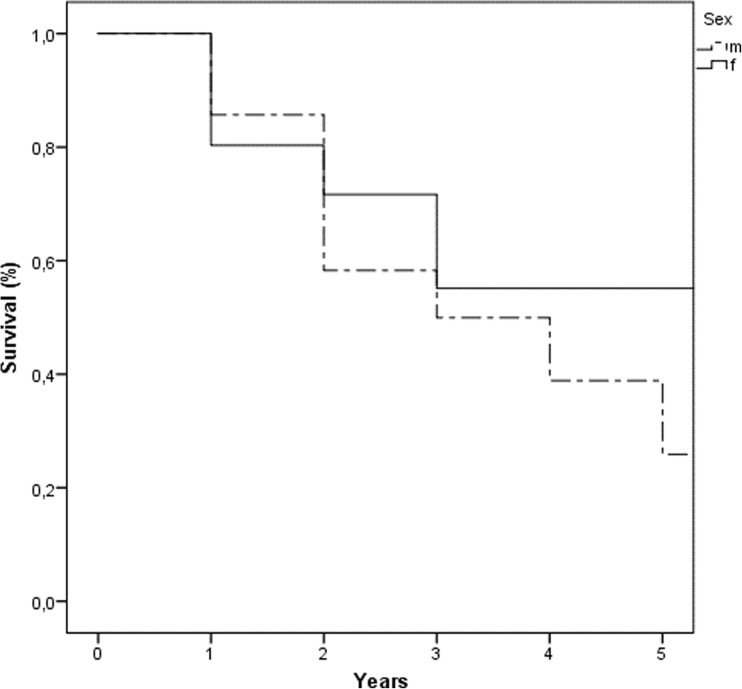
Gender-specific (m, male; f, female) overall survival (%)

**Fig. 3:**
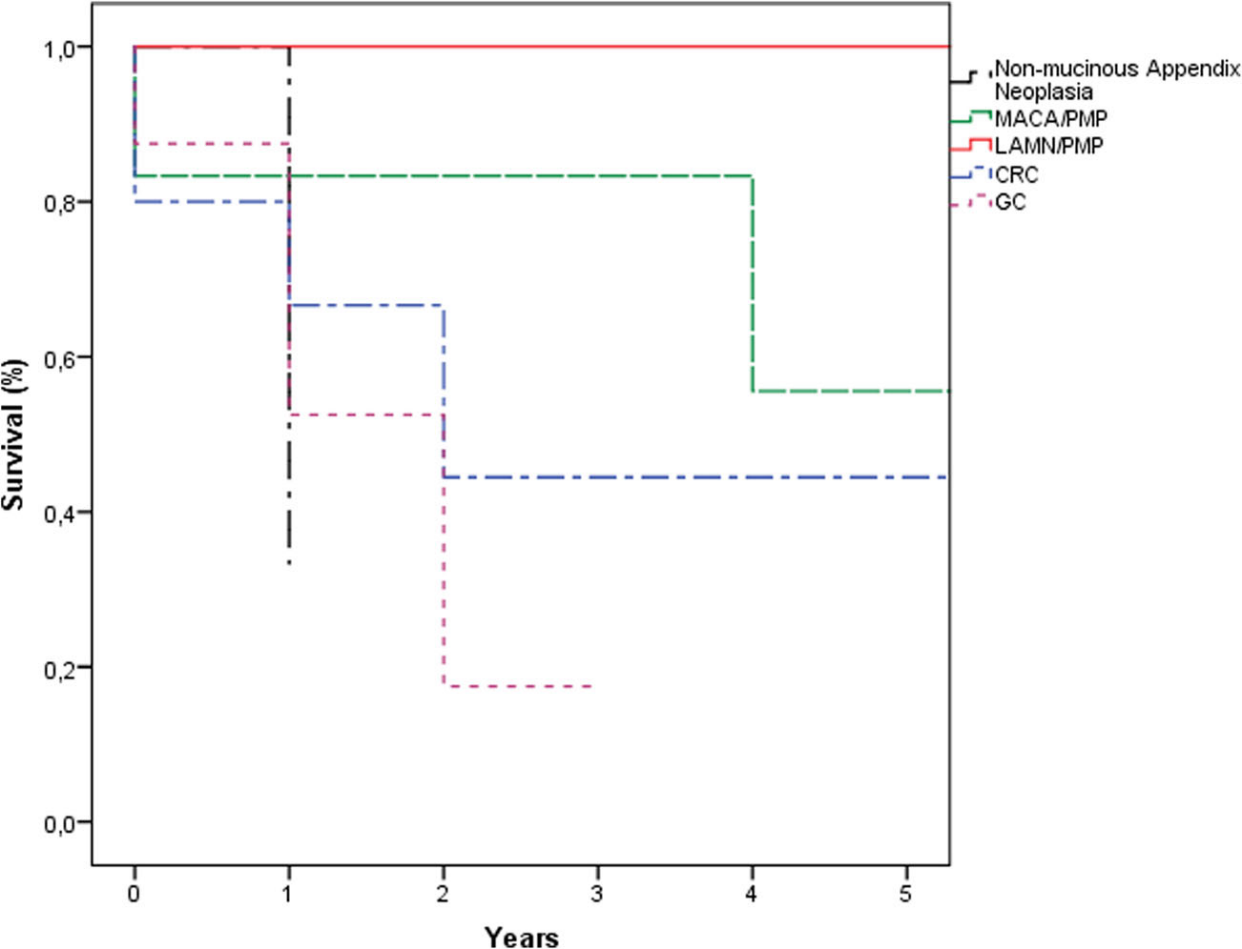
Overall survival (%)—various tumor entities (LAMN, low-grade appendiceal mucinous neoplasia; MACA, mucinous adenocarcinoma; CRC, colorectal cancer; GC, gastric cancer)

Overall, 5-year survival in our study population was 43% (Fig. 1) with a median survival of 39 months, 5-year disease-free survival was 33%. Itemized gender-specific, 5-year overall survival was 55% in women and 26% in men (Fig. 2). Overall, best outcome was seen in patients with PSM of mucinous appendix neoplasia, whereas patients with non-mucinous appendix neoplasia and gastric cancer primary showed worst overall survival (Fig. 3).

## Discussion

PSM is generally considered the terminal stage of a tumor disease with a very poor prognosis. Untreated, PSM can lead to death within several months and treatment in general comprises only palliative procedures, e.g., systemic therapy or palliative surgical interventions (feeding tubes, enteric bypass, stoma). Since the 1980s, the assumption that PSM is a generalized systemic metastatic spread shifted to it being a localized tumor spread similar to liver metastases (now defining the intraabdominal cavity as a localized compartment,^7^). Thus, new therapeutic strategies were developed with the objective of potential curation. The technique of complete cytoreductive surgery (CRS) combined with intraoperative hyperthermic chemotherapy (HIPEC) as described by Sugarbaker et al.^23^, ^24^ provides promising results in various tumor entities. In colorectal cancer, median OS can be drastically increased with CRS + HIPEC to up to 60 months with a 5-year OS of up to 51%.^7^,^12^,^25^ For PMP arising from mucinous appendix neoplasms, aggressive surgical debulking can achieve a 10-year OS of up to 30%,^26^–^29^ but with CRS + HIPEC median survival can be up to 16.3 years^4^ with a 5- and 10-year OS of 74 and 63%, respectively.^4^,^30^,^31^ Even for patients suffering from PSM arising from gastric cancer, which is generally associated with a very poor prognosis (median survival 1–3 months),^9^,^32^ CRS + HIPEC can increase median overall survival to up to 7–9 months^32^–^34^, or even 15 months if a CC score of 0/1 is realized. Studies investigating the effect of CRS + HIPEC on various tumor entities reported a median overall survival of 34 months with a 5-year overall survival of 37%.^18^ Itemized for the individual tumor entities, median survival was 30, 77, and 9 months for colorectal, appendix and gastric cancer, respectively.^18^

In our study population, median overall survival was 39 months with a 5-year overall survival of 43%, thus demonstrating results similar or slightly superior to those in the published literature. Interestingly, we were able to show a clear positive impact of female gender on survival, with 5-year OS and median OS being 55% and 67 months, respectively, as compared to a 5-year OS of 26% and a median OS of 34 months for male gender. Considering the relatively small number of patients included, itemizing overall and/or disease-free survival for every tumor entity included would not show statistical significance. But clearly, similar to the published literature, the impact on survival exerted by CRS + HIPEC for PSM arising from appendix neoplasms or CRC is superior to that for PSM from gastric cancer. Despite the heterogeneous patient population in our study with different tumor entities and a few very rare tumor entities like DSRCT, we recommend that generally no tumor entity with PSM should per se be excluded from the attempt of a curative treatment option with CRS and HIPEC. The decisive exclusion criteria however remain age, WHO status, PCI and estimated CCS. Of course, the application of HIPEC in some patients with tumors with very bad prognosis (e.g., pancreatic cancer) represents an experimental therapy and is only justifiable on explicit patient request after careful discussion in the multidisciplinary tumor board and meticulous patient informed consent.

Although promising results have been published for various tumor entities, CRS + HIPEC is still not an integral part of the treatment plan for patients suffering from PSM in numerous treatment facilities. Reasons may be various; most important factors for hesitation, in addition to a lack of resources and high costs, are the reported high perioperative patient morbidity and mortality rates. A large French registry addressed this concern, investigating a total of 1290 patients treated with CRS + HIPEC for PSM of different tumor entities at 25 different institutions. The mortality rate was 4.1%, main causes of death were multiorgan failure and septic shock. Morbidity rate, expressed as the result of major (grade III/IV) complications, was 33.6% with reoperation needed in 14% of patients. Most common complications were neutropenia, digestive fistulas or pneumonia, namely 13.3, 9.7, and 9.1%, respectively.^18^ Similarly, a Dutch trial reported a mortality rate of 3% with 34% morbidity.^35^ In our study population, 35 (35/60; 58.3%) patients developed any kind of complication, with 33.3% (20/60) grade III/IV complications. The most frequent grade III/IV complication was postoperative pleural effusion, likely caused by the HIPEC procedure and the extent of surgery performed at the diaphragm. Eleven (11/60; 20%) patients required reoperation, mostly due to postoperative hemorrhage.

The rather small number of patients with metachronous PSM in our study might be caused by the fact that for a long time medical oncologists, external surgeons, and medical practitioners did not consider these patients for curative treatment options. As a consequence, we did not have the possibility to offer CRS and HIPEC to patients with metachronous PSM. On the other hand, nowadays continuing education and positive cases of patients with CRS and HIPEC have succeeded and patients are transferred in time to our center already in the stadium of synchronous PSM. The subgroup analysis of patients with synchronous or metachronous PSM did not show a significant difference regarding postoperative complications or oncological outcome leading to the conclusion, that CRS and HIIPEC is feasible even in patients with metachronous PSM and supposed worse outcome.

Regarding the chemotherapeutic agents applied during HIPEC procedure, we used the recommendations of the Austrian Society of Surgical Oncology including five different chemotherapeutic regimens, which have changed during the study period. The actual ASSO standard (2015) recommends oxaliplatin as first choice for colorectal cancer, appendix neoplasia and PMP (alternative mitomycin C), cisplatin/doxorubicin as first choice for gastric cancer, peritoneal mesothelioma, and ovarian cancer.

When we started the program at our surgical department in 2006, our main focus was to build up a small team working with patients suffering from PSM, achieve strong expertise despite the limited number of patients, provide best possible conditions and realize optimal patient outcome. Addressing the inevitable learning curve, only six consulting surgeons at our institution performed CRS + HIPEC; in most cases two or more of these were present at every operation.^21^,^36^ This ensured not only similar intraoperative assessment of the extent of the PSM and evaluation of operability, but provided standardized OP techniques, cannula placement for HIPEC and reduced the duration of the procedure over time. By contrast, in order to include every PSM patient who was willing and potentially fit to bear with the extensive treatment regimen and thus reach high patient numbers, we defined from the beginning strict inclusion and exclusion criteria so as to achieve the best expertise in this surgical field and to not discredit a potential curative treatment, in full knowledge of the prevailing skepticism. Beside intensive preoperative patient evaluation, each patient’s medical chart was thoroughly assessed and discussed at our oncologic multidisciplinary team meeting. In each individual case, the recommendation for CRS + HIPEC called for us to focus on the likelihood of achieving complete cytoreduction (CC-0/1) and hence long-term disease-free survival, thus producing an intentionally highly selected patient population. Intense cooperation with our Department of Anesthesiology helped ensure thorough preoperative patient evaluation and preparation (e.g., anemia or malnutrition assessment and therapy), as well as intraoperative and early postoperative anesthesiology management. Patients treated with CRS + HIPEC need particular intraoperative fluid and coagulation management, mostly due to the duration of the procedure and the effects of administration of the hyperthermic intraperitoneal chemotherapy.^37^–^39^ In our patient population, intraoperative blood loss and therefore the need for PRBC transfusion was minor with a mean transfusion rate of 1.1 (0–7) concentrates in 20 (20/60; 33.3%) patients, although in 66.7% a multivisceral resection was performed. Coagulation disorders, evidenced in changes in the key blood parameters for clotting (INR/aPTT) or platelets, were seen more frequently with in particular 4.4 (0–20) FFP concentrates needed intraoperatively in 32 (32/60; 53.3%) patients. These results are consistent with the data published, delineating a need for transfusion (FFP or PRBC) in about 50% intraoperatively and in up to 28% postoperatively.^39^,^40^ Considering the potential early postoperative complications (hemodynamic, respiratory or coagulatory disorders, renal function impairment), all patients were directly transferred to our postoperative Intensive Care Unit and closely monitored for the first two postoperative days. As a result of all these perioperative precautions, we were able to keep morbidity low, even at the outset of our program and achieve a mortality rate of 0% in our study population, with no perioperative, in hospital, or 30-day mortality. An important lesson to learn for budding surgeons is the necessity of a small team of surgical oncologists experienced in multivisceral resection and interdisciplinary cooperation, who are able to manage severe postoperative complications.

In conclusion, since we started our program in 2006 at our surgical department, we have been able to confirm the feasibility of CRS + HIPEC for PSM of various tumor entities. Thanks to the intense collaboration enjoyed with various specialties and our definition of strict inclusion and exclusion criteria for patient selection, we were able to establish a well operating program for treating PSM in Austria. With acceptable morbidity rates that can be explained by the complexity of the procedure and no 30-day mortality, we succeeded in confirming the safety of this procedure at our surgical department. That said and noting the 5-year OS of 43% for PSM, CRS + HIPEC should be recognized as an important tool in advanced cancer therapy for the purpose of achieving curation in a select patient population.
